# A comparative study of the quality of care and glycemic control among ambulatory type 2 diabetes mellitus clients, at a Tertiary Referral Hospital and a Regional Hospital in Central Kenya

**DOI:** 10.1186/s13104-015-1826-0

**Published:** 2016-01-05

**Authors:** Shillah Mwaniga Mwavua, Edward Kiogora Ndungu, Kenneth K. Mutai, Mark David Joshi

**Affiliations:** Public Health Department, P.O.BOX 24218, Nairobi, 00502 Kenya; Division of Non-Communicable Diseases, Ministry of Health, Nairobi, Kenya; Partnership for Advanced Care and Treatment (PACT), Centre of Excellence, University of Nairobi, Nairobi, Kenya; Department of Clinical Medicine and Therapeutics’, School of Medicine, University of Nairobi, Nairobi, Kenya

**Keywords:** Diabetes mellitus, Glycemic control, Africa, Quality care

## Abstract

**Background:**

Peripheral public health facilities remain the most frequented by the majority of the population in Kenya; yet remain sub-optimally equipped and not optimized for non-communicable diseases care.

**Design and methodology:**

We undertook a descriptive, cross sectional study among ambulatory type 2 diabetes mellitus clients, attending Kenyatta National Referral Hospital (KNH), and Thika District Hospital (TDH) in Central Kenya. Systematic random sampling was used. HbA1c was assessed for glycemic control and the following, as markers of quality of care: direct client costs, clinic appointment interval and frequency of self monitoring test, affordability and satisfaction with care.

**Results:**

We enrolled 200 clients, (Kenyatta National Hospital 120; Thika District Hospital 80); Majority of the patients 66.5 % were females, the mean age was 57.8 years; and 58 % of the patients had basic primary education. 67.5 % had diabetes for less than 10 years and 40 % were on insulin therapy. The proportion (95 % CI) with good glycemic was 17 % (12.0–22.5 respectively) in the two facilities [Kenyatta National Hospital 18.3 % (11.5–25.6); Thika District Hospital 15 % (CI 7.4–23.7); P = 0.539]. However, in Thika District Hospital clients were more likely to have a clinic driven routine urinalysis and weight, they were also accorded shorter clinic appointment intervals; incurred half to three quarter lower direct costs, and reported greater affordability and satisfactions with care.

**Conclusion:**

In conclusion, we demonstrate that in Thika district hospital, glycemic control and diabetic care is suboptimal; but comparable to that of Kenyatta National Referral hospital. Opportunities for improvement of care abound at peripheral health facilities.

## Background

Diabetes mellitus is a global public health problem of epidemic proportions. An estimated 285 million people are now living with diabetes worldwide and this figure is expected to reach 440 million by year 2030, with the largest proportional increase occurring in developing countries [1]. Furthermore four out of every five people with diabetes live in developing countries, and most affected men and women are family bread winners [2].

During the past few decades a rapid increase in the prevalence of diabetes has been observed in low-and middle income countries, with high prevalence being reported even, among low income earner groups. This is well illustrated by two studies, that were done in the urban slums in Nairobi, The Kibera study [3], which demonstrated that there was a high prevalence of DM yet low screening rates in this previously unstudied slum, and the Viwandani and Korogocho study [4], which demonstrated that Diabetes was on the rise among the poor. In the developing countries, not enough is being done to provide the least expensive lifesaving diabetes drugs, for optimal long term glycemic control, and provide quality comprehensive care [5]. Health care systems are predominantly geared towards communicable diseases and not enough attention is being accorded to non-communicable diseases [6].

Studies indicate that treatment and control of Diabetes can be highly cost effective because medical care expenditures are avoided when therapy defers or delays the development of complications [7]. In Sub Saharan Africa, evidence indicates that much of the death and disability related to diabetes can be prevented with cost effective interventions [8].

Glycosylated hemoglobin recommended by the American Diabetes Association as the preferred and effective measure of glucose control and a target of less than 7.0 % is advised for most patients. It is useful in assessing the effectiveness of therapy and guiding the therapeutic decisions and as such is a useful measure of quality of care.

Barriers to quality diabetic care include lack of a definite policy addressing chronic illness, limited resources, restricted spectrum of care, and lack of information to the public and psychological factors. Emphasis in care has been on drug therapy, but omitting components such as education, nutrition counseling and psychosocial support [9]. These challenges are compounded by regionally unique social, cultural and economic issues [10]. Studies have also shown that clients perceive psychological issues as the most important barrier to diabetes care; [11] besides the strictness of the diabetes regimen, including dietary, exercise, self-glucose monitoring, clinician review, and medication activities [12].

In Kenya the National Diabetes strategy 2010–2015 estimates Diabetes prevalence at 3.3 % with a projected rise to 4.5 %, in 2025, translating to 1.8 million Kenyans living with Diabetes [13]. With her per capita GPD estimated at $1200 a year most diabetics even with subsidies are unable to afford diabetes health care,that is the cost of drugs, transportation and laboratory test [14]. Hence making diabetes care unaffordable to those who really need it.

Peripheral public health facilities where the majority of the affected Kenyan population seek health care services continues to be sub optimally equipped and not geared to non-communicable diseases care. This has led to widely held perception that person with diabetes can only get quality medical care from the tertiary health facilities, which has contributed to an over bearing, self-referral, patient load at the limited number of tertiary facilities. Services at tertiary facilities are costly and is further compounded by the other indirect client costs including transport [15].

Provision of quality diabetic care at peripheral facilities is best suited towards making care and management reachable to majority of Kenyans who need it.

The objective of this study was therefore to compare quality of diabetes care provision, level of glycemic control and direct patients cost, at a tertiary referral hospital to that at a regional hospital in Central Kenya.

## Methods

This was a comparative cross-sectional study conducted at Kenyatta National Referral Hospital (KNH) and Thika District Hospital (TDH) in Kenya. KNH is situated in the Kenya’s capital city, Nairobi, approximately 2 km to the west of the city Centre, KNH is one of two national referral hospital and is also a teaching hospital for the University of Nairobi’s College of Health Sciences. The Hospital has a bed capacity is 1800 and 4800 members of staff. KNH runs specialized diabetics clinic managed by a team of specialist, endocrinologists, physicians, graduate resident doctors, nutritionists, diabetic educators, medical assistants, and nurses. Approximately 400 diabetic clients are seen in a week at clinics that run daily excluding weekends. TDH is a primary health care facility located 80 km to the east of Nairobi City. The health facility has a bed capacity is 250 and 500 members of staff. TDH runs a single weekly diabetic outpatient clinic once that attends to approximately 80 clients. The team of clinicians includes medical officers, medical assistants, nursing practitioners and a nutritionist. The study was carried out among ambulatory Type II DM clients, receiving care and follow-up at the selected facilities. Inclusion criteria was clients above 18 years of age, diagnosed at least 6 months earlier and on glucose lowering therapy for at least 3 months. Patients diagnosed to have type 1 diabetes mellitus (WHO criteria), pregnant females and patients diagnosed or documented to have psychological or mental disorders were excluded.

### Sampling, recruitment and data collection

The clients who met the inclusion criteria and provided written consent were systematically sampled, by recruiting every third client. Structured questionnaires were administered by two study investigators (SMM and ENK) assisted by two trained research assistants, to capture data on socio-demographic characteristics, co-morbidities and diabetes diagnosis and management variables. Patients were questioned on direct out of pocket expenses incurred per clinic visit, clinic appointment intervals and patients perceived accessibility to and affordability of care and treatment. Furthermore they were asked to indicate whether they were satisfied or not with the level of current care provided. Level of adherence to oral treatment was established using the Morisky scale giving three categories of high, intermediate and low adherence. A finger-prick blood sample was obtained from each patient for HbA1c measurement after each interview. HbA1c was measured onsite using Bayer A1CNow^®^, a portable, easy-to-use device capable of providing HbA1c results in 5 mins. This had the advantage of communicating the results to the patient immediately. Quality control for the HbA1c tests was done by drawing 1.0 ml venous blood samples from every 20th patient and transported on the same day to an external accredited laboratory for the test [16, 17].

### Ethical considerations

The study received approval from the Kenyatta National Hospital (KNH)/University of Nairobi (UON) Ethics and Research Committee. Permission to collect data was obtained from the Administration of KNH and the TDH.

### Statistical analysis

Statistical analysis was performed using SPSS version 18.0. Chi square test was used to test associations between categorical variables with an alternative of Fisher’s exact test used where the numbers were small. Comparison of means was done using Student’s t test and Mann–Whitney U test to compare medians. Odds ratio was used to estimate the relative risk of good glycemic control in association with categorical independent variables. Pearson correlation was done to compare point of care HbAIC results with the laboratory Hba1c result. All statistical tests were performed at 95 % confidence level and statistical significance considered at p value of less or equal to 0.05.

## Results

Between August and October 2012 we screened 210 diabetic clinic clients, excluded ten and enrolled 200, who were included in the final analysis; 120 (60 %) were from Kenyatta National Hospital (KNH) and 80 (40 %) from Thika District Hospital (TDH). Reasons for exclusion included: two clients who were pregnant and below 18 years of age, three had a diagnosis of type 1 diabetes, and three had been enrolled into care for less than 3 months.

### Demographic characteristics

The mean age of the clients was 57.8 years (SD 12.3 years) of which 66.5 % were females. Majority (58 %) of the clients had achieved primary level of education, 99 % were of Christian faith and 80 % married (Table 1). Age, sex and education distribution between the two facilities were not significantly different.

**Table 1:** Socio-demographic characteristics

Variable	Overall n (%)	KNH n (%)	TDH n (%)	P value
Mean age (SD)	57.8 (12.3)	56.9 (12.8)	59.0 (11.3)	0.351
Gender				
Female	133 (66.5)	77 (64.2)	56 (70.0)	0.247
Highest level of education				
Primary	116 (58.0)	63 (52.5)	53 (66.3)	0.054
Secondary	84 (42.0)	57 (47.5)	27 (33.7)	
Religion/faith				
Christian	198 (99.0)	118 (98.3)	80 (100.0)	0.518
Marital status				
Married	160 (80.0)	98 (81.7)	62 (77.5)	0.734
Single	8 (4.0)	4 (3.3)	4 (5.0)	
Separated/divorced/wido wed	32 (16.0)	18 (15.0)	14 (17.5)	

### Diabetes mellitus characteristics

Among the entire sample 67.5 % of clients had a duration of diabetes diagnosis of less than 10 years, however this proportion was significantly higher among TDH clients (TDH 80 %; KNH 59.2 %; P = 0.002) (Table 2). Diabetes had been diagnosed at screening exercise in 47.5 % and on illness evaluation in 51.5 %. Hypertension was the most common co-morbidity recorded in 57 %. Insulin therapy was used by 40 % of the clients with a significantly larger proportion among KNH patients (KNH 63.3 %; TDH 5 %; P = <0.001).

**Table 2:** Disease and treatment characteristics

Variable	Overall n (%)	KNH n (%)	TDH n (%)	P value
Duration of diabetes				
≤10 years	135 (67.5)	71 (59.2)	64 (80.0)	0.002
≥10 years	65 (32.5)	49 (40.8)	16 (20.0)	
Diabetes and co-morbidities				
Hypertension	114 (57.0)	66 (55.0)	48 (60.0)	0.484
Asthma	7 (3.5)	4 (3.3)	3 (3.8)	1.000
Stroke	4 (2.0)	2 (1.7)	2 (2.5)	1.000
Arthritis	14 (7.0)	5 (4.2)	9 (11.3)	0.054
Diabetes diagnosis				
Routine hospital check up	13 (6.5)	7 (5.9)	6 (7.5)	0.640
Screened during medical camp	95 (47.5)	57 (48.3)	38 (47.5)	1.000
Sick with symptoms of diabetes	90 (45.0)	54 (45.8)	36 (45.0)	1.000
Not stated	2 (1.0)			
Current treatment/advice				
Insulin	80 (40.0)	76 (63.3)	4 (5.0)	<0.001
Special prescribed diet	187 (93.5)	109 (90.8)	78 (97.5)	0.061
Advice/treatment to lose weight	129 (64.5)	91 (75.8)	38 (47.5)	<0.001
Frequency of blood sugar monitoring				
Daily/ weekly	38 (19.0)	23 (19.2)	15 (18.9)	0.780
Monthly	59 (29.5)	33 (27.5)	26 (32.5)	
Don’t test	103 (51.5)	64 (53.3)	39 (48.8)	

### Glycemic control

Good glycemic control defined on basis of HBAIC was detected in 17 % of the study patients (95 % CI 12.0–22.5) and did not differ between the two facilities (Table 3). [KNH 8.3 % (95 % CI 11.5–25.6); TDH 15 % (95 % CI 7.4–23.7); P = 0.539].

**Table 3:** Factors associated with glycemic control

Variable	Glycemic control		OR (95% CI)	P value
	Good (HbA1c<7)	Poor (HbA1c≥7)		
Age	59.6 (12.4)	57.4 (12.3)	–	0.351
Gender				
Female	22 (64.7)	111 (66.9)	0.9 (0.4–2.0)	0.808
Male	12 (35.3)	55 (33.1)	1.0	
Highest level of education				
None	5 (14.7)	29 (17.5)	1.0	
Primary	14 (41.2)	68 (41.0)	1.2 (0.4–3.6)	0.754
Secondary	10 (29.4)	51 (30.7)	1.1 (0.4–3.7)	0.829
Tertiary	5 (14.7)	18 (10.8)	1.6 (0.4–6.4)	0.493
Duration of diabetes				
≤10 years	26 (76.5)	109 (65.7)	1.7 (0.7–4.0)	0.220
More than 10 years	8 (23.5)	57 (34.3)	1.0	
Frequency of self monitoring				
Daily/weekly	3 (8.8)	35 (21.1)	1.0	
Monthly	11 (32.4)	48 (28.9)	2.7 (0.7–10.3)	0.141
Don’t test	20 (58.8)	83 (50.0)	2.8 (0.8–10.1)	0.100
Frequency of clinic visits				
Weekly/fortnightly	1 (2.9)	5 (3.0)	1.0	
Monthly	22 (64.7)	113 (68.1)	1.0 (0.1–8.7)	1.000
6 months	11 (32.4)	48 (28.9)	1.1 (0.1–10.8)	1.000
Accessibility				
Easy	31 (91.2)	154 (92.8)	0.8 (0.2–3.0)	0.724
Difficult	3 (8.8)	12 (7.2)	1.0	
Affordability				
Affordable	19 (55.9)	104 (62.7)	0.8 (0.4–1.6)	0.460
Not affordable	15 (44.1)	62 (37.3)	1.0	
Satisfaction				
Satisfied	33 (97.1)	165 (99.4)	0.2 (0.0–3.3)	0.312
Not satisfied	1 (2.9)	1 (0.6)	1.0	
Compliance				
High adherence	24 (70.6)	77 (46.4)	2.8 (1.2–6.2)	0.010
Intermediate/low adherence	10 (29.4)	89 (53.6)	1.0	
Diabetic drugs availability				
Yes all the drugs	31 (79.5 %)	8 (20.5 %)	1.0	0.637
Yes some drugs are available	120 (82.8 %)	25 (17.2 %)	1.2 (0.5–3.0)	0.221
No	15 (93.8 %)	1 (6.3 %)	3.9 (0.4–33.8)	

Comparison between the HbA1c values between point of care A1C and laboratory HbA1c using the paired t test showed no significance. [POC 8.3 (1.2) QC 7.8 (1.2) P = <0.211] (Table 4).

**Table 4:** HbA1c paired t test comparison

	POC	EQA	P value
HbA1c	8.3 (1.2)	7.8 (1.2)	0.211

On bivariate and multivariate analysis (Table 5) compliance to medication was the only variable associated with good control (adjusted OR 3.2, 95 % CI 1.4–7.6 (Table 6)with no facility differential effect. Half of the clients (50.5 %) had a high level adherence to diabetes medication, with a higher proportion among KNH clients (KNH 45 %; TDH 58.8 %; P = 0.057). Overall, 51.5 % of the clients did not do out of clinic blood sugar monitoring (self-monitoring or otherwise) and this was not significantly different between KNH and TDH. However, 29.5 and 19 % of the clients reported having their blood monitored on a monthly and weekly basis respectively.

**Table 5:** Bivariate and multivariate analysis: predictors of good control

Variable	OR (95 % CI)	P value
Age	1.02 (0.98–1.05)	0.386
Gender		
Female	1.0 (0.4–2.4)	0.972
Male	1.0	
Highest level of education		
Below primary	0.8 (0.3–1.8)	0.519
Above secondary	1.0	
Duration of diabetes		
≤10 years	1.0	
More than 10 years	0.5 (0.2–1.3)	0.138
Frequency of self-monitoring		
Daily/weekly	1.0	
Monthly	2.6 (0.6–10.7)	0.176
Don’t test	3.3 (0.8–12.6)	0.086
Appointment intervals clinic visits		
Weekly/fortnightly	1.0	
Monthly	1.0 (0.1–10.0)	0.998
6 months	1.2 (0.1–12.7)	0.877
Compliance		
High Adherence	3.2 (1.3–7.6)	0.009
Intermediate/low adherence	1.0	
Diabetic drugs availability		
Yes all the drugs	1.0	
Yes some drugs are available	0.8 (0.3–2.1)	0.588
No	0.3 (0.0–2.5)	0.247

**Table 6:** Compliance of the patients to medication

Variable	Overall	KNH	TDH	P value
		n (%)	n (%)	
Morisky scale				
High adherence	101 (50.5)	54 (45.0)	47 (58.8)	0.057
Intermediate /low adherence	99 (49.5)	66 (55.0)	33 (41.2)	
Would you visit any other health facility for the management of your diabetes?				
Yes	32 (16.0)	25 (21.2)	7 (8.8)	0.020
No	168 (84.0)	93 (78.8)	73 (91.3)	

### Measures of quality of care

Clinic appointment and evaluations, majority (85 %) of clients at both hospitals reported regular attendance at their scheduled clinic appointments. The intervals of scheduled clinic appointments ranged from fortnightly to six monthly; 67.5 % were scheduled three monthly and 29.5 % six monthly (Table 7). The proportion of clients receiving one to three monthly appointments was 92.6 and 50.8 % at TDH and KNH respectively. However 46.7 % of clients in KNH were scheduled at six monthly intervals.

**Table 7:** Quality of care

Variable	Overall	KNH n (%)	TDH n (%)	P value
How frequent are clinic visits				
Week/fortnightly	6 (3.0)	3 (2.5)	3 (3.8)	<0.001
Between 1–3 month	135 (67.5)	61 (50.8)	74 (92.6)	
6 months	59 (29.5)	56 (46.7)	3 (3.8)	
Attend clinical appointments as scheduled				
Yes	170 (85.0)	102(85.0)	68 (85.0)	1.000
No	30 (15.0)	18 (15.0)	12 (15.0)	
Tests during routine check up				
RBS	199 (99.5)	119 (99.2)	80 (100.0)	1.000
BP	195 (97.5)	115 (95.8)	80 (100.0)	0.159
HbA1c	5 (2.5)	5 (4.2)	0 (0.0)	0.159
Urinalysis	33 (16.5)	9 (7.5)	24 (30.0)	<0.001
WT	74 (37.0)	28 (23.3)	46 (57.5)	<0.001
Ever missed medications in past 6 months?				
Yes	46 (23.0)	29 (24.2)	17 (21.3)	0.631
No	154 (77.0)	91 (75.8)	63 (78.8)	
Are Diabetic drugs always available				
Yes all the drugs	39 (19.5)	33 (27.5)	6 (7.5)	<0.001
Yes some drugs are available	145 (72.5)	84 (70.0)	61 (76.3)	
No	16 (8.0)	3 (2.5)	13 (16.3)	
IF DM drugs unavailable, source?				
Chemist	159 (98.8)	85 (97.7)	74 (100.0)	0.500
Don’t buy	2 (1.2)	2 (2.3)	0 (0.0)	

The clinical evaluations routinely made at clinic visits included, random blood sugar in 99.5 %, blood pressure (BP) measurement in 97.5 %, weight in 37 % and urinalysis in 16.5 % (Table 7). HBAIC was done in 2.5 % of clients; 4.2 % at KNH and none at TDH. Urinalysis, weight measurement were significantly more frequently done at TDH compared to KNH (TDH 30.5 %; KNH 7.5 %; P = <0.001 respectively). Proportion of clients given lifestyle change advice counseling was: 93.5 %; special prescribed diet (KNH 90.8 %; TDH 97.5 %; P = 0.061) and 64.5 %, on weight loss (KNH 75.8 %; TDH 47.5 %; P < 0.001).

### Drug availability

Overall, 92 % of the clients reported that drugs for diabetes were available in both facilities. Availability of all diabetic drugs was reported more frequently in KNH (27.5 %) than in TDH (27.5 %; 7.5 %; P = <0.001) while none availability was more common at TDH than at KNH (16.3 %; 2.5 %; P < 0.001).

### Cost of care 

Clients’ means of transport to clinic was predominantly public transport. However, use of private means was significantly higher at KNH (12.5 %) and a (Table 8) higher proportion of TDH (11.3 %) clients walked to clinic. Clients at KNH spent significantly higher amount of money in Kenya shillings (Kshs) on transport (median value in Kshs 400.0 KNH; 160 TDH), consultation (median Kshs KNH 550.0; TDH 100.0) and on oral glycemic agents (median Kshs KNH 1000.0; TDH 300). The cost of insulin was the same at the two facilities.

**Table 8:** Cost of care

Variable	Overall	KNH n (%)	TDH n (%)	P value
Are you usually accompanied when coming to the clinic?				
Yes	70 (35.0)	48 (40.0)	22 (27.5)	0.069
No	130 (65.0)	72 (60.0)	58 (72.5)	
Which means of transport do you use when attending the clinic?				
Public means	167 (83.5)	99 (82.5)	68 (85.0)	0.641
Private means/walking	33(16.5)	21 (17.5)	12 (15.0)	

Variable	Overall	KNH	TDH	
How much do you spend on transport per clinic day?				
Median (IQR)	250 (165–600)	400 (200–1000)	160 (100–200)	
How many clinics do you visit per month?				
Mean (SD)	1.0 (0.2)	1.0 (0.1)	1.1 (0.2)	
How much do spend on transport to the clinic every month?				
Median (IQR)	280 (185–600)	400 (200–1000)	160 (100–220)	
Consultation fees				
Mean (SD)	550 (100–550)	554.0 (35.3)	105.6 (50.3)	
Insulin				
Mean (SD)	370 (350–400)	439.4 (201.9)	456.7 (75.1)	
Medicine fees				
Median (IQR)	700 (300–1500)	1000 (600–2000)	300 (200–400)	

### Accessibility, affordability and satisfaction

The proportion of clients reporting that they were satisfied with current care, found it easily accessible, and affordable was 92.5 % (KNH 90.8 %; TDH 95 %; P = 0.273), 61.5 % (KNH 53.3 %; TDH 73.8 %; P = 0.004) and 90 % respectfully (Table 3). Majority (84 %) of the clients reported that they would not transfer to another health facility for management of their diabetes illness (TDH 91.3 %; KNH 78.8 %: P = 0.020).

## Discussion

We set out in this study to compare the quality of out-patient follow-up care offered to persons with type 2 diabetes mellitus at a referral and regional level hospital in Kenya; on the premise widely held by patients that higher quality of care is offered at tertiary facilities. However, we report that the level of glycemic control, as documented by HbA1c levels, is poor and comparable at both facilities. Less than 20 % of clients were well controlled. Drug compliance levels were also low and compliance was the only multivariate predictor of poor control. Low compliance levels are reported despite more than three quarter of clients reporting honoring their clinic appointments. Insulin availability and cost were the same but oral hypoglycemic drugs were more frequently unavailable at the peripheral center.

As markers of quality of care offered, clients at the regional facility were more likely to have a clinic driven routine urinalysis and weight done, and were accorded shorter clinic appointment intervals. Direct costs incurred by patient were half to three quarter lower at the regional facility. Compared to tertiary facility clients, regional facility clients reported greater affordability and satisfactions with care offered, and were less inclined to transfer care to other centers.

We are not aware of studies in Kenya comparing diabetic care between centers. However, studies on level of glycemic control at Kenyatta National Hospital have been documented; a cross-sectional descriptive study done in 2012 at the outpatient diabetes clinic of KNH reported that only 20 % of patients had ever done at least one HbA1c check [18].

In another study done in 1998 in KNH they found that Most patients (71 or 68 %) had very poor long-term glycemic control with an HbA1c level >10.0 %, concluding that the majority of ambulatory diabetic patients attending the out-patient diabetic clinic had poor glycemic control [19].

In a 2002 retrospective study on review of clinical records that was performed in Kwa Zulu natal district in South Africa and Random blood glucose, hemoglobin A1c (HbA1c) and urine albumin/creatinine ratio assayed it Acceptable glycemic control (HbA1c < 2 % above normal population range) was found in only 15.7 % of subjects (95 % confidence interval (CI): 11.4–20.8 %). Mean HbA1c was 11.3 %, therefore concluding that care and control of diabetes in this rural community was sub optimal [20].

A prospective cohort study done in 2008 at Mekelle Hospital in northern Ethiopia concluded that in this severely resource- limited areas, glycemic control amongst diabetic clients was very poor, and attributed this to scattered populations, shortage of drugs and insulin and lack of diabetes team care as major contributing major factors [21].

In another cross sectional, descriptive study done at the University of Benin Teaching Hospital, Benin City, a tertiary health facility in Nigeria, between June and December 2004, it showed that many of the persons with diabetes mellitus in Benin city still had poor glycemic control similar to previous reports [22].

A study in Finland concluded that the follow-up of most diabetic patients—including type 1 diabetes—can be organized in primary health care with the same quality as in secondary care units. The centralized primary care of type 1 diabetes is less, costly and requires fewer specialist consultations [23].

Another study in China concluded that the overall status of glycemic control was unsatisfactory. Although, patients at tertiary hospitals appeared to have better control than those at primary or secondary hospitals [24, 25].

### Implications of findings

Our findings point to the need for policy makers to focus their attention on strategies that address quality of care at both regional and tertiary facilities. Targeted interventions at improving care and facilities at peripheral centers will help patient’s access better health care and thus achieve good glycemic control with the attended benefits of complication and cost savings. Decentralization of diabetes care to county, sub-county and health centers is therefore important to enable success in care and management of diabetes at lower care levels and only have complicated cases referred to tertiary facilities.

### Strengths and limitations

To the best of our knowledge, our study is the first to compare quality of regional and tertiary center diabetic care in Kenya. A further strength is use of the guideline validated and recommended measure of HbA1c for glycemic control. Though we relied on qualitative non-standardized patient dependent information for some of the measure of quality of care, for purpose of comparison, we have no reason to believe that any bias would be differential.

## Conclusions

In conclusion we demonstrate that regional glycemic control and diabetic care is suboptimal but comparable to that of a national tertiary center. However, several measures of diabetic care at the regional facility were better and more affordable and satisfying to clients. Furthermore measures to improve diabetes care at regional care facilities are readily evident.
